# The Young Dentist Has Come

**Published:** 1902-01-15

**Authors:** 


### THE YOUNG DENTIST HAS COME.

The new graduate is now among us in abundance. He comes with the idea that he has mastered all the deep mysteries of dentistry, and, like Pare, feels that nothing further can ever be discovered. His egotism is to be excused, for he undoubtedly has learned more theoretical and scientific dentistry than we have had an opportunity of absorbing. He looks on us as out-of-date old fogies, and we gaze upon him in amazement for his presumption in pitting his book lore against the clinical wisdom gleaned through many years of arduous practice.

He settles near us, and some of our patients go to him. He is somewhat more enthusiastic than we in making his operations painless, and will at times slight his work rather than have it painful. He seems to be winning from us, and human nature seems to demand that we feel "sore," and show it; soon there is an open rupture, and the people say that the "old doctor" is jealous.

There are redeeming features in the matter. If he gets some of our best patients he is coerced into attending many whom we are glad to be rid of. He wins practice, but we are still busy. If he glories in his perfect knowledge of the latest developments in bacteriology we may tell him some facts learned by sad experience. He has unbounded faith in humanity, and we may be somewhat softened by his zeal. We need each other, and it is a mistake if we drift apart when we should be mutually attracted. The old doctor may learn much from the younger competitor, and he should be glad to do so; the younger man should give due respect to the older dentist's mature judgment. We like to cultivate the acquaintance of young dentists; while they may smile at our ignorance and antiquity, we are amused at their freshness, so both parties are entertained. While they laugh at us, we are learning from them; while we laugh at them, we try to give them some of the fruits of our long experience. The older man is too pompously dignified; the younger man is too impudently impudent, and the breach is widened.

Let us get together; learn from each other; bear with each other's frailties, and get along well together.

Let the old man learn that he is growing old, that he has not fully kept pace with dental progress, that younger men must eventually supersede him. Let the young man learn that the older practitioner views him somewhat as an interloper ; let him be satisfied to humor the old man's whims ; let him respect the matured experience of arduous years. Let both be charitable, and let both be honest and work for the best interests of themselves and their patients.—Editorial in *Dental Clippings*.

